# A consistent muscle activation strategy underlies crawling and swimming in *Caenorhabditis elegans*

**DOI:** 10.1098/rsif.2014.0963

**Published:** 2015-01-06

**Authors:** Victoria J. Butler, Robyn Branicky, Eviatar Yemini, Jana F. Liewald, Alexander Gottschalk, Rex A. Kerr, Dmitri B. Chklovskii, William R. Schafer

**Affiliations:** 1 Division of Cell Biology, MRC Laboratory of Molecular Biology, Cambridge Biomedical Campus, Francis Crick Avenue, Cambridge CB2 0QH, UK; 2 Janelia Farm Research Campus HHMI, Ashburn, VA 20147, USA; 3 Buchmann Institute for Molecular Life Sciences and Institute of Biochemistry, Goethe University, Max-von-Laue-Strasse 15, 60438 Frankfurt, Germany

**Keywords:** *Caenorhabditis elegans*, locomotion, gait adaptation, phase-shift, biophysics, muscle activity

## Abstract

Although undulatory swimming is observed in many organisms, the neuromuscular basis for undulatory movement patterns is not well understood. To better understand the basis for the generation of these movement patterns, we studied muscle activity in the nematode *Caenorhabditis elegans. Caenorhabditis elegans* exhibits a range of locomotion patterns: in low viscosity fluids the undulation has a wavelength longer than the body and propagates rapidly, while in high viscosity fluids or on agar media the undulatory waves are shorter and slower. Theoretical treatment of observed behaviour has suggested a large change in force–posture relationships at different viscosities, but analysis of bend propagation suggests that short-range proprioceptive feedback is used to control and generate body bends. How muscles could be activated in a way consistent with both these results is unclear. We therefore combined automated worm tracking with calcium imaging to determine muscle activation strategy in a variety of external substrates. Remarkably, we observed that across locomotion patterns spanning a threefold change in wavelength, peak muscle activation occurs approximately 45° (1/8th of a cycle) ahead of peak midline curvature. Although the location of peak force is predicted to vary widely, the activation pattern is consistent with required force in a model incorporating putative length- and velocity-dependence of muscle strength. Furthermore, a linear combination of local curvature and velocity can match the pattern of activation. This suggests that proprioception can enable the worm to swim effectively while working within the limitations of muscle biomechanics and neural control.

## 1. Introduction

Efficient movement through an environment requires an animal to select a locomotion pattern that is appropriate to the physical nature of its surroundings. How an animal's nervous system produces the rhythmic neuromuscular activity that is responsible for generating such patterns, for switching between them and for adapting them to perturbations in the external environment, is a question that has received considerable attention [1–3].

For the nematode *Caenorhabditis elegans*, the relationship between body wall muscle activity and worm posture depends on the environment of the worm. When crawling on a firm substrate, wild-type *C. elegans* display a characteristic sinusoidal movement pattern with a wavelength shorter than the body [1,4,5]. However, when swimming in liquid of low viscosity, *C. elegans* display a bending posture with a longer wavelength and higher frequency [5,6]. Note that we use *crawling* to indicate movement on the interface between a firm substrate and a low-viscosity fluid (on agar or agarose in air), while *swimming* refers to locomotion in a fluid (low or high viscosity).

Despite the identification of a small subset of neurons that control worm locomotion [7], it is currently unclear whether crawling and swimming locomotion result from two distinct gaits produced by functionally separate neural circuits, or from the modulation of one gait produced by a single neural circuit. In support of the existence of distinct neural circuits, several studies have shown that crawling and low-viscosity swimming behaviours can be described by distinct kinematics [1,5,8]. Body bending during low-viscosity swimming is faster and has a longer wavelength and lower curvature than when crawling. Furthermore, the biogenic amines dopamine and serotonin have been shown as necessary and sufficient for switching between swimming and crawling locomotion patterns [9]. This study also observed bimodal kinematics when external resistance on the worm was increased via changes in viscosity, pressure applied by compression and substrate resistance applied to certain portions of the worm's body by magnetic pull.

However, a number of other studies have reported that gradually increasing the external mechanical resistance on a swimming worm induces a gradual decrease in bending wavelength and bending frequency [1,6,10,11], supporting the single modulated gait hypothesis. Differences in locomotion pattern could arise largely from the mechanical consequence of different external environments on output of a single neuromuscular program [12], and a gradual transition is hypothesized to allow the worm to maintain propulsive thrust through a range of resistive environments [1].

Theoretical models of sinusoidal locomotion in crawling snakes and nematodes [13–15] attempt to relate muscle activity to body curvature by considering the forces acting on the animal during locomotion. During swimming in water, the drag forces on the worm's body in liquid are predicted to be small compared with the elastic forces that the worm must overcome to bend its body, indicating that maximal muscle activity should coincide with maximal body curvature [1]. During crawling or high-viscosity swimming, the frictional forces (drag in fluids, and a combination of drag and plastic deformation on agar gels) dominate and overcoming these forces is of key importance. Each body segment contributes propulsive thrust depending on its angle of attack, the angle between the direction of overall forward movement and the tangent vector to the body segment [1,13]. Propulsive thrust and drag is therefore maximal when approaching a region of increasing body curvature rather than at the maximal curvature itself, and decreases rapidly with a drop in angle of attack. As such, it is predicted that maximal muscle activity should occur before maximal body curvature. The magnitude of this phase-shift could be as much as 90° of the bending cycle [13], though models using the angle of attack and biomechanical parameters of undulatory movement predict approximately 60° phase difference for swimming in high-viscosity fluids [1].

The neuromuscular anatomy of *C. elegans* does not suggest an obvious way in which muscle activation could occur at widely varying phases depending on external load. Motor neuron processes synapse in a repeating pattern with approximately fixed spacing [16]. Although dynamic or nonlinear activity could generate a variety of phase-shifts from this neuromuscular architecture, the simplest hypothesis is that muscular activation occurs a fixed distance in advance of a particular curvature, and that passive mechanical properties of the system are largely responsible for the change in locomotion pattern.

To investigate the biomechanics underlying gait adaptation and distinguish between predictions of force generation, it is desirable to record the activity of muscles in worms in a variety of external conditions and exhibiting a variety of locomotion patterns. Although electrophysiology in swimming and crawling animals is not practical, calcium imaging is possible [17–19]. We therefore built an imaging system capable of performing calcium imaging on moving animals at adequate spatial and temporal resolution, and used the calcium indicator GCaMP3 [20] to follow body wall muscle activity under a variety of external conditions. These recordings reveal that during both swimming with various external loads and crawling, worms maintain approximately the same phase offset between maximum activation and maximum curvature. These results suggest the importance of proprioceptive feedback in the control of both swimming and crawling and provide important empirical constraints on mechanistic models of *C. elegans* locomotion.

## 2. Material and methods

### 2.1. Strains

Strains were maintained at 22°C, using standard protocols [21]. Alleles used in this study were: N2 (wild-type), *sma-1*(*e30*) V, *sma-6*(*e1482*) II, *dpy-11*(*e207*) V, *lon-2*(*e678*) X and *lon-3*(*e2175*) V.

### 2.2. Molecular biology

The generation of body wall muscle plasmids *Pmyo-3::GCaMP3-SL2-tagRFP-T* and *Pmyo-3::GFP-SL2-tagRFP-T* was carried out as follows. The *myo-3* promoter region was obtained from plasmid pDEST-myo-3p [22], a gift from H. Kuroyanagi. A 2.3 kb NotI/BamHI fragment was ligated to worm codon-optimized GCaMP3 and tagRFP-T (DNA2.0 Inc., USA), gifts from L. Looger, within the vector pSM (C. Bargmann, Rockefeller University), a derivative of vector pPD49.26 (A. Fire, Stanford University). A 870 bp XmaI/EcoRI GFP fragment was ligated in place of the GCaMP3 within the vector pSM, to generate a control construct.

### 2.3. Microinjections, integrations and crosses

Transgenic lines were obtained by injection of plasmids *Pmyo-3::GCaMP3-SL2-tagRFP-T* and *Pmyo-3::GFP-SL2-tagRFP-T* at a concentration of 50 ng μl^−1^, along with 100 ng μl^−1^ 1 kb DNA Ladder (Invitrogen, Life Technologies Ltd, UK) into N2 wild-type worms. Both constructs express in the body wall muscle and vulval muscle of hermaphrodites. Integrated lines AQ2953 *ljIs131*[*Pmyo-3::GCaMP3-SL2-tagRFP-T*] and AQ2954 *ljIs132* [*Pmyo-3::GFP-SL2-tagRFP-T*] were obtained by UV irradiation. Integrants underwent 10 generations of singling and backcrossing before use in experiments. Mutant lines carrying integrated arrays were generated by first crossing N2 males into each integrated line. Fluorescent males carrying the integrated array were then crossed into mutant lines. Three F_1_ worms heterozygous for the mutation and integrated array were singled. F_2_ worms were singled from these plates if they were fluorescent and displayed the phenotype of interest.

### 2.4. Tracking system

A Dino-Lite Pro AM413T USB camera (Dino-Lite Digital Microscopes, The Netherlands) is used for automated worm tracking. Tracking lighting is provided by a red LED (M627L1 627 nm 500 mW, Thorlabs, USA) controlled by a T-Cube LED driver (LEDD1B, 0–1200 mA 12 V, Thorlabs) and filtered at 633–647 nm by a Brightline single-band bandpass filter (FF01–640/14–25, Semrock, Inc., USA). The red light passes through a BrightLine single-edge dichroic mirror (FF580-FDi0125 × 36, Semrock, Inc.) that reflects the beam through the calcium imaging objective lens and onto the sample plane. T-LSR075A Motorized Linear Slides (Zaber Technologies Inc., USA) support a 0.5 inch × 18 inch × 12 inch aluminium breadboard stage (custom made by Janelia Farm Instrument Design and Fabrication, Janelia Farm Research Campus, USA) and give automated *x*–*y* movement. Movement of the linear slides is controlled through Worm Tracker v. 2.0 Software v. 2.0.3.1. The stage supports an iXon 885 EMCCD calcium imaging camera (Andor Technology Plc., USA).

### 2.5. Calcium imaging

Fluorescence excitation is provided by two optically pumped semiconductor lasers. A Sapphire 488 nm laser (Coherent, Inc., Germany) provides excitation for green fluorescence and a Sapphire 561 nm laser (Coherent, Inc.) provides excitation for red fluorescence. Each laser beam is free-space coupled into a high-power fused silica single-mode fibre-optic patchcord (QSMJ-3AF, 3A-488–3.5/125–3AS-1 Oz Optics Ltd, USA) via a FibrePort Collimator (PAF-X-7A, *f* = 7.5 mm, 350–700 nm, Thorlabs). Each fibre output is connected to a lens tube (SM1L20, Thorlabs) via a fibre adaptor cap (S120-FC, Thorlabs). Laser excitation is delivered to the sample plane using a two dichroic mirror system that reflects both laser beams up through the calcium imaging objective lens. A BrightLine single-edge dichroic mirror (FF510-Di01–25 × 36, Semrock, Inc.) reflects the 488 nm laser and transmits the 561 nm laser onto a second BrightLine single-edge dichroic mirror (FF580-FDi0125 × 36, Semrock, Inc.). This second dichroic mirror reflects both laser beams onto a pellicle beamsplitter (BP108, uncoated 8 : 92 R : T, Thorlabs) positioned between the tube lens and the 2× objective.

Emitted fluorescence is recorded through an MVX Plan Apochromat 2× objective (Olympus America, Inc., USA, working distance 20 mm, numerical aperture 0.5). The fluorescence image is split into a green channel and a red channel by an Optosplit II image splitter (Cairn Research Ltd, UK) containing a BrightLine single-edge laser-flat dichroic mirror (Di01-R561–25 × 36, Semrock, Inc.) that transmits light wavelengths above 561 nm. The two channels are each projected onto one half of the EMCCD camera chip. Green emission is filtered at 498 nm to 553 nm wavelengths by a Brightline single-band bandpass filter (FF03–525/50, Semrock, Inc.) and red emission is filtered at 581 nm to 619 nm wavelengths by a Brightline single-band bandpass filter (FF01–600/37–25, Semrock, Inc.). The presence of an additional StopLine quad-notch filter (NF01–405/488/561/638–25 × 05.0, Semrock, Inc.) in the green channel light path blocks excitation light from the lasers. One hundred millimetre and 250 mm focal lenses positioned between the output end of the optic fibre patch cord and dichroic mirrors project a concentrated laser spot onto the worm at the sample plane. A tube lens (MVX-TLU from MVX10 Macroview Microscope, Olympus America, Inc.) between the Optosplit II beamsplitter and the 2× objective brings parallel light rays from the 2× objective together at the intermediate image plane. The total magnification produced by the combination of the objective lens and tube lens was calculated as 3.95.

Calcium imaging was performed using Andor Solis Imaging Software (Andor Technology Plc., USA). All images were obtained at a rate of 26 Hz, with an exposure time of 0.01147s (to minimize blurring during fast head and tail movements during swimming in low viscosity) and 2 × 2 pixel binning. Output images from this software were saved as 32-bit dat files and imported into ImageJ for further processing.

### 2.6. Locomotion assays

All locomotion assays were performed on L4 stage worms. Crawling assays were performed by picking single worms to a 100 μl NGM pad. A single worm was picked to the pad, tracked and imaged between the NGM pad and a coverslip. Crawling movement was unhindered with S-shaped dorsal and ventral bends propagating from the head towards the tail over time. Swimming assays were carried out in 20 µl M9 buffer droplets with 0–80% dextran w/w (Sigma, USA, Cat. No. 95771). Movement in the *z*-axis was minimized by placing the worm between a microscope slide and a coverslip, with an additional coverslip acting as a spacer. Swimming movement was unhindered with C-shaped dorsal and ventral bends propagating from the head towards the tail over time.

### 2.7. Image processing

A flat-field correction was applied to each image sequence to correct for uneven laser illumination across the field of view. Muscle calcium imaging analysis was performed using a custom MATLAB script. The analysis locates the edges of the worm in both the green and red channels using the Canny edge detection algorithm. These edges are used to create a mask over the worm, and this mask is filled to give a solid worm. The mask is used to find a first approximation of the midline of the animal and locate the two ends corresponding to the worm's head and tail. The bright bands of muscle on each side of the midline are identified and used to fix the position of the midline. A constant-speed spline fit is used to improve the location of the midline. The background around the worm is located, measured and used for background correction of fluorescence measurements. A bleed-through correction is also performed to correct for bleed-through of green fluorescence into the red channel. The fluorescence ratio is calculated as the background-corrected green fluorescence divided by the background—and bleed-through—corrected red fluorescence. Pixels corresponding to the muscle of the worm are projected from the outline onto the midline of the worm to give a fluorescence and curvature measurement at a particular point along the worm body (1 = head, 100 = tail). This results in the correction for compression and stretching of the muscle on body bending. Fluorescence measurements are normalized to the mean fluorescence. Maxima in curvature traces correspond to ventral body bends and minima in curvature traces correspond to dorsal bends. The wavelength of the animals was determined by digitizing the body, reducing it to a curve and finding the distance between two consecutive complete bend points (extrema of the curve). Distance between bends was measured in different time points and averaged across them. The digitized body curve was filtered with a (causal) square wave filter to reduce noise in its acquisition and facilitate bend detection.

### 2.8. Measurement of GCaMP3 fluorescence kinetics

Vulval muscle imaging was carried out as previously described [23], with some modifications. Adult worms were immobilized with 2-octyl cyanoacrylate glue (Dermabond topical skin adhesive, Ethicon, USA) on an agarose pad (2% agarose in 10 mM HEPES, pH 7.1, 13 mOsm kg^−1^) and covered with the same HEPES buffer. A Zeiss Axioskop 2 upright microscope equipped with a Hamamatsu Orca R2 CCD camera, and a Uniblitz Shutter (Vincent Associates, USA) was used to acquire the images with Metavue v. 7.6 (Molecular Devices, USA) at 15fps.

The GCaMP3 fluorescence decay time was measured by superimposing eight fluorescent decays that each occurred after a quick relaxation visible as muscle motion. The best-fit exponential (0.45 + 0.55e^−t/1.57^) gave the GCaMP3 decay time constant as 1.57 s, with a decay half-life of 1.09 s. Measuring the time delay between peak muscle contraction (taken as the maximal distance of the muscle from its resting position) and the peak fluorescent signal, gave an estimate of the GCaMP3 fluorescent latency as 22 ± 11 ms.

To estimate the time delay between body wall muscle depolarization and a change in the GCaMP3 signal, we combined whole cell patch clamp recordings with simultaneous Ca^2+^imaging. Electrophysiological recordings from immobilized and dissected *C. elegans* were essentially performed as described previously [24]. After dissection, cells were treated for 8 s with 0.5 mg ml^−1^ collagenase (Sigma, Germany) in *C. elegans* Ringer's (CR; 150 mM NaCl, 5 mM KCl, 5 mM CaCl_2_, 1 mM MgCl_2_, 10 mM glucose, 15 mM HEPES (pH 7.35), 340 mOsm kg^−1^) and washed with CR. The bath solution was CR, the pipette solution was 115 mM K-gluconate, 25 mM KCl, 0.1 mM CaCl_2_, 5 mM MgCl_2_, 1 mM BAPTA, 10 mM Hepes, 5 mM Na_2_ATP, 0.5 mM Na_2_GTP, 0.5 mM cAMP, 0.5 mM cGMP, pH 7.2, with approximately 320 mOsm kg^−1^ KOH. Recordings were done on an inverted fluorescence microscope (Zeiss, Axioskop 2 FS plus) equipped with a HBO 50 lamp, a 40× water immersion objective (Zeiss Achroplan) and a GFP Filter (Zeiss FS10). Body wall muscles were initially clamped to a holding potential of −30 mV using an EPC10 amplifier (HEKA, Germany) and Patchmaster software (HEKA, Germany). During recordings, we initiated a 100 ms voltage jump in the holding potential via the amplifier from −30 to +30 mV, similar to values that have been previously recorded for action potentials in *C. elegans* [25]. Images were obtained with a CCD camera (Photometrics Coolsnap HQ 2, Roper Scientific) using µ-Manager 1.4 open source software (http://www.micro-manager.org/). Illumination protocols were triggered via the amplifier using a TTL pulse and images were recorded at 10 Hz. Image processing and analysis was done using ImageJ v. 1.47 software (http://rsb.info.nih.gov/ij/) generating ROIs for the patched muscles. Data were calculated to obtain (*F* − *F*_0_)/*F*_0_.

### 2.9. Estimation of muscle activity from calcium traces

#### 2.9.1. Sparse fit method

We assumed that calcium reported in the muscle was determined by a first-order decay to baseline plus influx from electrical activity:
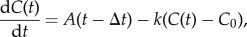
where *C* is the output of the calcium indicator (ratio or intensity), *A* is the time-course of activity of the muscle, Δ*t* is the latency of muscular contraction compared to the onset of calcium signal, *k* is a decay constant and *C*_0_ is the calcium indicator output at rest. We therefore converted to a difference equation
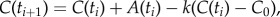
where time steps were at the camera frame rate (i.e. 33 ms). In body wall muscles, we observed decays with a time constant of down to approximately 1 s, and found that motionless worms had a ratio of GCaMP to tagRFP-T of approximately 0.2 (range 0.17–0.22). Since we were primarily concerned with relative activity, we simply set *k* = 1/30 (equivalent to 1 s^−1^) and *C*_0_ = 0.2. To estimate activity, we then solved for *A*(*t_i_*):
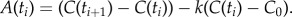


This estimate was highly noise-sensitive (data not shown), so we performed unconstrained optimization (fminsearch/fminunc in MATLAB v. 7.12) on *A*(*t*) to fit *C*(*t*) minimizing the cost function
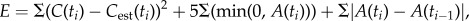
where *C*_est_ is the estimate of *C* from application of the values of *A* through the difference equation for *C*. The second term is to penalize relatively non-physiological ‘negative activity’. Although the L1 norm in the third term favours relatively stepwise-constant predictions of *A* rather than a sinusoidal activation, a stepped approximation of a sinusoid has no phase offset from an actual sinusoid, so is suitable for making determinations of relative phase. Without more definitive experimental confirmation of muscle activity—from electrical recording, for instance—we do not believe it is practical to definitively distinguish sinusoidal from stepped activation on the basis of calcium imaging.

#### 2.9.2. Amplitude distribution method

Electrophysiological recordings indicated that the response of a calcium indicator to a brief excitation of muscle was best fit by a two-exponential process
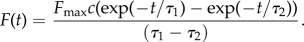


Convolving a particular pattern of activation with this function would result in both a phase-shift and a decrease in maximum amplitude. To find the time constants *τ*_1_ and *τ*_2_, we used only amplitude data and used these values to estimate the phase lag induced by the filtering.

In particular, we first split all our calcium measurements into short-period (less than 1.5 s) and long-period (over 1.5 s) groups; the short-period data were used to compute the scaling constant *F*_max_ while the long-period data were used to find a best fit for *τ*_1_ and *τ*_2_ (unconstrained optimization with fminsearch in MATLAB v. 7.12). The expected phase lag for each period was then computed and subtracted from the measured phase-shift.

### 2.10. Phase-shift measurements

Phase-shift measurements were performed in MATLAB using the cross-correlation function *xcorr*. The period of the sinusoidal movement was calculated by averaging the distance between adjacent maxima in the cross-correlation sequence. The phase-shift for ventral muscle activity was calculated as the displacement of the central maxima from a phase lag of 0. The phase-shift for dorsal muscle activity was calculated as the displacement of the central minima from a phase lag of 0. A positive phase-shift indicates maximal muscle activity occurring before maximal body curvature. A negative phase-shift indicates maximal muscle activity occurring after maximal body curvature. For high-viscosity recordings, these measurements were made directly on the calcium and curvature traces. For low-viscosity recordings, peaks in curvature were found and the corresponding calcium traces were scaled and averaged; this in general can lead to blurring of the calcium signal but was necessary to have adequate signal-to-noise ratio. When high-viscosity data were treated similarly, average phase-shift agreed closely with direct measurement (data not shown).

### 2.11. Estimation of muscle activation

To predict the phase relationship between maximal bending and maximal required torque on our worms, we used eqn 7 from [1], recast as
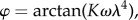
where *K* = *C_N_*/*b*(2π)^4^. Since a value for the coefficient of viscous drag *C_N_* was not reported, we digitized figures 3*a,b* and 5*c* and fit for *K*, which resulted in excellent agreement (less than 1.5° discrepancy for any measurement). As we used younger animals than were used in [1], both *C_N_* and the internal elasticity *b* were presumably different, so we considered values of *K* within one order of magnitude of that in [1], producing a range of possible force–viscosity relationships.

To estimate required effort, we considered the case where muscle force generation is dependent on both velocity and length. These relationships can be complex and depend on the biophysical details of force generation in the muscle [26], but for this study we adopted a simple linear scaling as in [12]. In brief, muscle strength is affine in both velocity and length, with rapidly contracting or highly contracted muscles weaker than stationary or rest-length ones. Since body posture is approximately sinusoidal, we estimated muscle strength as
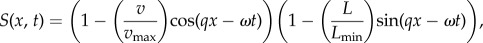
where *x* is the position along the body, *t* is time, *q* = 2*π*/*λ* specifies the wavelength, *ω* the (angular) frequency of oscillation, *v*_max_ the theoretical maximum velocity (at which the muscle could produce zero force) and *L*_min_ the maximum contraction (where *L* = 0 at zero curvature). Since the animal's body approximately follows a sinusoid, we considered that the effort exerted by the muscle would have to be proportional to the force required and inversely proportional to the muscle strength:
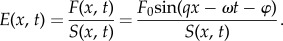


We assumed calcium signals were proportional to effort and performed an exhaustive search of parameter space for (*v*_peak_/*v*_max_) and (*L*_nadir_/*L*_min_) between 0 and 1, where *v*_peak_ is the fastest observed velocity (at 0% dextran) and *L*_nadir_ the greatest contraction (at 50% dextran). The phase of peak effort was determined numerically for each condition, and choices for parameters were scored by least-squared difference from the phase advance at each condition determined by calcium imaging. Finally, we also considered that force generation may be delayed from muscle activation; we performed parameter sweeps from 0 to 200 ms delay.

To investigate whether proprioceptive feedback could in principle generate the observed activation profile, we considered linear combinations of curvature and velocity in the idealized (sinusoidal) case. Since curvature and velocity sensors could each be anywhere along a motor neuron's process, we again performed a parameter sweep for sensing locations (spacing up to 0.4 of the animal's body length, with the anterior-most sensing location at most 0.2 of a body length anterior to the site of excitation), sign (positive meaning activated by contraction, negative meaning activated by stretch) and relative strength of curvature and velocity.

### 2.12. Statistical analysis

Standard statistical tests were performed using MATLAB functions. Correlation coefficients were calculated using the MATLAB function *corrcoef*. A value of +1 indicates perfect positive correlation. A value of −1 indicates perfect negative correlation. Two-sample *t*-tests performed a *t*-test of the null hypothesis that two sets of data are independent random samples from normal distributions with equal means and equal but unknown variances, against the alternative that the means are not equal. Confidence intervals were estimated with bootstrap sampling of the data. Specifically, we resampled the population of animals with replacement, then for each animal resampled recordings of that animal if there was more than one, and then re-ran the analysis routines we were using. Two hundred resampled datasets were used in each case.

## 3. Results

### 3.1. Development of a whole-worm tracking and calcium imaging system

**Figure 1.**
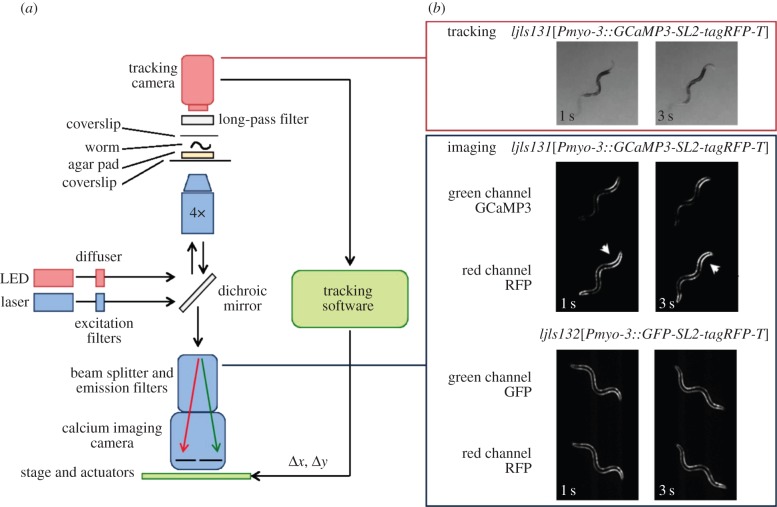
Tracking calcium imaging microscope. (*a*) Schematic of the calcium imaging set-up. (*b*) Image sequences from each camera: worm tracking and re-centring (above) and ratiometric calcium imaging (below). Note increased fluorescence on contracted side of body bends in GCaMP but not control channel (arrowheads).

We developed a whole-worm tracking and calcium imaging system, in which high-resolution single-worm tracking was combined with measurement of body wall muscle activity. We achieved this by modifying Worm Tracker v. 2.0 [27,28] for calcium imaging. An EM-CCD camera with calcium imaging optics and a USB camera for tracking were both placed on a motorized stage (figure 1*a*). This allowed tracking without moving the animal, thus preventing sloshing and other bulk movement of the liquid in which the animal was swimming. We selected filters and LED illumination such that we could monitor both green and red fluorescence channels as well as monitor behaviour with deep red illumination (figure 1*b*).

We expressed GCaMP3 [20] in body wall muscle to monitor calcium; to correct for motion artefacts, calcium-insensitive tagRFP-T was co-expressed with GCaMP3. We also assayed control worms expressing calcium-insensitive GFP in place of GCaMP3 to check for residual artefacts. As expected, we observed strong modulation of GCaMP3 fluorescence with body bends, but little modulation of GFP or tagRFP-T (figure 1*b*).

**Figure 2.**
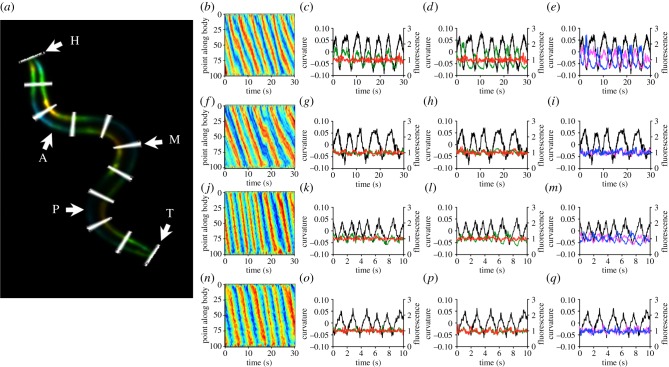
Body wall muscle activity in freely crawling and swimming worms. (*a*) Image processing analysis. The body divided into 100 segments (H = head, segment 1; T = tail, segment 100; every 10th segment is highlighted in white) and midline curvature and dorsal and ventral fluorescence intensity is measured. Segments 25, 50 and 75 are selected to represent anterior, midbody and posterior muscle activity (A, M and P, respectively). Body wall fluorescence is coloured by curvature (reds, positive; blues, negative). (*b*) Body curvature kymograph during crawling. Red bands represent ventral bends; blue bands represent dorsal bends. (*c*) Ventral GCaMP (green) and tagRFP-T (red) fluorescence with curvature (black, ventral contraction positive). (*d*) As (*c*) but dorsal fluorescence. (*e*) Comparison of dorsal (blue) and ventral (magenta) fluorescence. (*f–i*) As (*b–e*) but for a control animal expressing GFP in place of GCaMP3. (*j–m*) Curvature kymograph during swimming (*j*), curvature and ventral fluorescence (*k*), curvature and dorsal fluorescence (*l*), and curvature with ventral and dorsal GCaMP3 fluorescence (*m*) in an animal swimming in 40% dextran, following figure 2*b–e*. (*n–q*) Same, but for a control animal expressing GFP in place of GCaMP3.

To quantify the relationship between posture and muscle activity, we measured midline curvature and dorsal and ventral fluorescence in 100 segments from the worm's head to its tail (figure 2*a*); from among those we selected segments 25, 50 and 75 as representative of anterior, midbody and posterior muscle activity, respectively. These points were chosen specifically to avoid the head region where force generation and bend initiation are confounded. We first imaged muscle activity during crawling on a firm substrate (2% agarose). Curvature kymographs showed alternation between ventral and dorsal bends, with smooth propagation along the body from head to tail. Since phases of dorsal, midline and ventral curvature did not differ appreciably from each other (electronic supplementary material, figure S3 and data not shown), we used midline curvature for all comparisons. Consistent with previous results that used cameleon as a calcium indicator [5], GCaMP3-expressing strains showed calcium transients in both dorsal and ventral muscles that were out of phase with each other and locked to curvature (figure 2*b–e* and electronic supplementary material, figure S1). By contrast, GFP control strains showed similar curvature but no fluorescence signal (figure 2*f–i*). We then imaged muscle activity in Newtonian fluids as previously described [1], with qualitatively similar results (figure 2*j–q*). Thus, our tracking system was able to follow calcium transients in freely moving worms under a variety of conditions.

### 3.2. GCaMP3 fluorescence is a filtered representation of calcium signals

**Figure 3.**
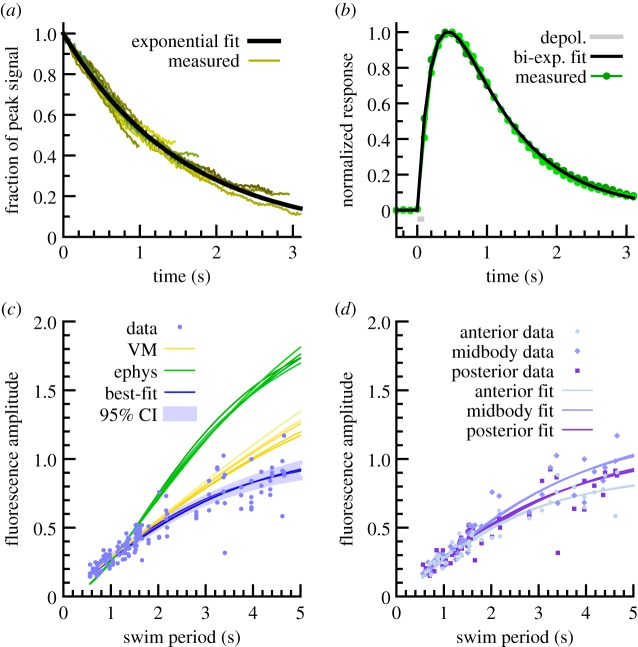
Estimation of signal filtering from GCaMP3 kinetics. (*a*) GCaMP3 fluorescence decays observed during rapid relaxation of vulval muscle, along with best single-exponential fit (time constant 1.57 s). (*b*) GCaMP3 signal induced by electrical activation of muscle (indicated in grey). Green, individual trials (*n* = 2). Black, best two-exponential fit, e^−*t*/0.25^ − e^−*t*/0.88^ (time units seconds). (*c*) Fluorescence amplitude as a function of locomotion period across all viscosities, plus crawling. Blue dots, raw data (*n* = 48 animals, three body positions each). Green lines, relationship expected from parameters found from electrophysiology with different assumptions about shape of underlying activation. Yellow lines, relationship expected from vulval muscle derived parameters. Blue lines, relationship expected with best fit of two-exponential filtering function, e^−*t*/0.05^ − e^−*t*/0.50^. Confidence interval determined by bootstrap resampling of data. (*d*) Amplitude–period relationship for anterior, midbody and posterior sites, plus best fits to each; differences are not statistically significant at this sample size.

Despite the improved kinetics of GCaMP3 [20], the fluorescence response of all genetically encoded calcium indicators remains slow relative to the rapid calcium transients that accompany channel openings and cell depolarization [29,30]. In order to accurately compare the timing of muscle activity relative to body curvature, it is therefore necessary to know the kinetics of fluorescent onset and decay in *C. elegans* muscle. We measured the time delay between maximal muscle contraction and maximal GCaMP3 fluorescence, as well as the decay time for GCaMP3 fluorescence, during spontaneous contraction of vulval muscle. The maximal fluorescence was coincident with maximum contraction to within the temporal resolution of our measurements (0.03 s), and we estimated the decay time as 1.57 s by superimposing decays in fluorescence (figure 3*a*). Thus, we concluded that phase lag may be considerable.

To gain a better estimate of phase lag in body wall muscle, we recorded calcium transients from dissected worms while providing electrical stimulation. The resulting fluorescence change was well-fit by a biexponential of the form exp(−*t*/*τ*_1_)−exp(*t*/*τ*_2_) with a fast (rise) time constant of 0.25 s, a slow (decay) time constant of 0.88 s (figure 3*b*), and a peak 0.44 s after the excitation. This late peak suggested that for swimming, phase delays would result in a signal completely out of phase with contraction, which was not what we observed. We hypothesized that calcium handling may be upregulated in active muscle [31,32], which could lead to increased calcium binding and clearance and thus shorter time constants in active worms. Filtering will in general decrease the amplitude of signals as frequency increases, so we compared observed peak-to-peak fluorescence signals at different swimming frequencies and compared to the amplitude trend predicted from our electrical measurements (figure 3*c*). We found that the predicted amplitude fit the data poorly even when restricted to fitting short periods and did not track long-period amplitudes at all. The values estimated from vulval muscle produced a better but still unsatisfactory fit. We then found optimal values of the time constants T1 and T2, assuming that the animal would efficiently use the dynamic range of its neurons and therefore that maximal calcium influx would be similar across locomotion at different rates. Bootstrap analysis indicated that a variety of parameters produced decent fits, with a mean best fit of 0.05 and 0.50 s for fast and slow time constants, respectively (peak at 0.13 s; figure 3*c*). Fits were similar at anterior, midbody and posterior sites (figure 3*d*). We concluded that in active muscle, GCaMP3 fluorescence was a filtered indicator of calcium influx, but that the impact of filtering could be quantitatively assessed.

### 3.3. Muscle activity–body curvature phase-shifts are similar during crawling and swimming

**Figure 4.**
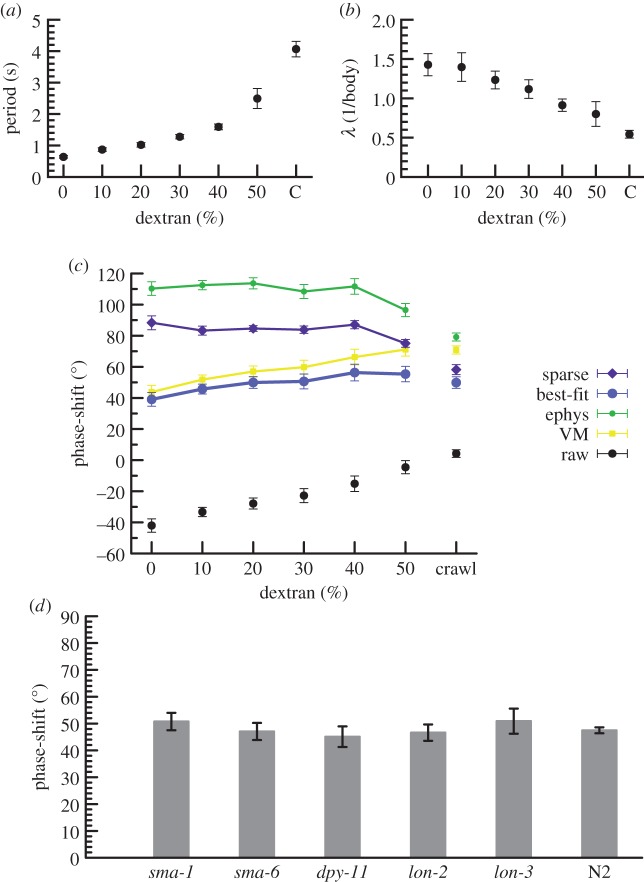
Locomotion parameters and muscle activity–body curvature phase-shifts. (*a*) Period of oscillation of animals crawling over an agarose gel (C) or swimming in solutions with different percentages of dextran to increase viscosity. Error bars, s.e.m. *n* ≥ 4 samples per data point. (*b*) Wavelength of body bends as a fraction of body length during sinusoidal movement. (*c*) Estimated phase-shift between peak curvature and peak muscle activation (positive indicates activation leading curvature), using corrections suggested from vulval muscle recordings (yellow squares), electrophysiology (green circles), best-fit of amplitude–period relationship (blue circles) or filtering with sparsity (low number of changes) constraint (purple diamonds). Black circles indicate uncorrected measurements. Error bars, s.e.m. (*d*) Estimated phase advance in various mutants during crawling using the best-fit method from (*c*).

We first assessed how the animals' locomotion pattern changed under conditions of different viscosity by using kymographs to calculate the period and wavelength of sinusoidal movement. Consistent with previous studies [1,5,10], we found that period increased and wavelength decreased with increasing viscosity (figure 4*a,b*).

By detecting the relative position of curvature and calcium peaks, we were able to calculate phase-shifts for each oscillation. We noted that maximal activity occurred slightly earlier in posterior body locations than anterior (electronic supplementary material, figure S2), consistent with shifts found in larger organisms [33–37]. It is also possible that differential levels of filtering create an appearance of a phase advance, as the differences in best-fits, though not significant, are large enough to obscure the anterior–posterior phase advance.

We then corrected the measured phase-shifts by the lag expected due to filtering, by predicting calcium flux assuming an intermediate level of filtering (single-exponential decay, 1.0 s) plus a sparsity condition that penalized large or repeated changes in predicted calcium flux, or by simply assuming the parameters measured in VMs or with electrophysiology. In all cases, and contrary to theoretical predictions of force [1], the phase-shift remained consistent across all viscosities (figure 4*c*). The constant varied by method; for the best-fit method the phase-shift had a predicted median value of 49.9° (95% CI of 46.5–53.3) and showed non-zero but minimal dependence on viscosity (±9.4°, 95% CI of 4.8–14.5). In addition, when using our best estimate for filtering of calcium transients, the phase relationship was also similar for crawling (figure 4*c*). Collectively, this indicates a common mechanism for muscle activation across different environmental conditions.

To test the impact of body morphology on phase relationship, we recorded calcium transients in mutants with small bodies (*sma-1*, *sma-6*), with squat bodies (*dpy-11*) and with long bodies (*lon-2*, *lon-3*). Two of these mutants showed bending periods longer than wild-type (N2, *T* = 4.0 ± 0.2 s, *n* = 14 versus *sma-1*, *T* = 5.7 ± 0.8 s, *n* = 6, *p* < 0.05 and *lon-2*, *T* = 5.6 ± 0.4 s, *n* = 7, *p* < 0.01; *p*-values uncorrected for multiple comparisons). Nonetheless, in all cases the phase-shifts were similar to wild-type (figure 4*d*), further indicating that the worm's neuromusculature generates muscle activity at a fixed phase ahead of contraction, largely independent of mechanical factors.

### 3.4. Reconciliation of muscle force with muscle activity

To investigate how muscle activation and predicted muscle force could have such large differences, especially at low viscosity, we considered four factors that are important for force generation. First, worms in our system could have somewhat different internal elasticity or experience somewhat different viscous drag than reported in [1], owing to differences in experimental configuration and age of animals. Second, force generation is likely to be delayed: brief illumination of animals expressing channelrhodopsin-2 in muscle indicates a latency of the order of 80 ms [24]. Third, the biophysics of motor proteins on myosin lead to force–velocity and force–length curves [38]; although these curves are not known for *C. elegans* and can be quite complex in general, we considered a simple linear relationship as this produced decent results in a neuromechanical model [12].

**Figure 5.**
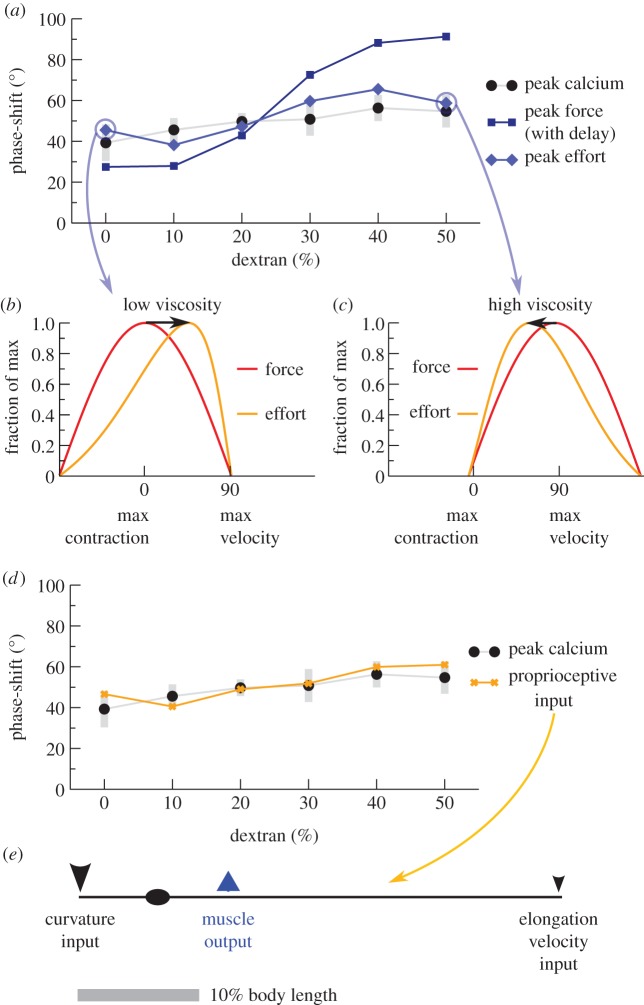
Force–activity agreement with biophysically limited muscle. (*a*) Muscles with both force–velocity and force–length curves (light blue diamonds) are predicted to require maximal activation at phases similar to that inferred from calcium imaging (best-fit method, black circles; grey bars indicate 95% bootstrap CI), while a simple lag between activation and force generation cannot (dark blue squares). Peak effort is computed with two constraints: *v*_peak_/*v*_max_ = 0.8 (fastest observed rate of contraction was 80% of the maximum biophysically possible under zero load), and *L*_nadir_/*L*_min_ = 0.62 (greatest observed contraction was 62% of the maximum possible, neglecting body elasticity). (*b,c*) Possible mechanism of phase conservation from biophysical limits. (*b*) At low viscosity (0% dextran), muscles near maximum possible velocity when animals are near-straight, requiring effort (orange) that peaks in advance of force (red). (*c*) At high viscosity (50% dextran), force (red) peaks near maximum velocity but effort (orange) peaks with a relative phase delay where muscles are more contracted. (*d*) A linear combination of locally sensed stretch and velocity (orange Xs) can match the observed phases of activation (best-fit method, black circles), suggesting that proprioception would be sufficient to generate the pattern. (*e*) Illustration of the sensing and output pattern of neurons in (*d*). Arrow size indicates relative strength of input. Note that many other similar configurations also match observed phases.

We performed an exhaustive search, varying all four parameters, to find combinations where the muscle activation required for predicted force generation was in close agreement with calcium imaging. Contractile latency alone produced a poor fit (figure 5*a*); latency with altered drag and elasticity was somewhat better but still poor (data not shown). By contrast, a combination of force–velocity and force–length relationships yielded good agreement, both with no latency (figure 5*a*) and across a variety of latencies and elasticities (data not shown). Intuitively, this is because when worms are moving fastest in low viscosity, they must exert extra effort to maintain speed at the faster but less contracted part of the cycle (figure 5*b*), while at high viscosity the speed is reduced and extent of contraction increased, requiring extra effort towards the more contracted but slower part of the cycle (figure 5*c*).

Motor neurons are known to have oscillatory activity [18,19], but the precise phase relationship at different viscosities has not been reported. To investigate whether individual motor neurons could have the information to generate the pattern of muscle activation we observe, we tested whether a weighted sum of curvature or velocity information gathered from various points along the process of B-class motor neurons, which are required for propagation of curvature [39], could yield the observed activation profile. For idealized motor neurons roughly consistent with actual anatomy (as taken from fig. 4.13 in [40]), the answer was yes (figure 5*d*, best-fit method; others data not shown): motor neurons potentially have access to proprioceptive information that enables them to generate a near-constant phase activation. In particular, best-fit phase correction suggests a model where slowly adapting receptors at the anterior tip of B-class motor neurons are activated by contraction and rapidly adapting receptors at the posterior tip are more weakly activated by increasing stretch (figure 5*e*); however, it is probable that there are many other possibilities when less simplistic models of sensing and activation are considered.

Taken together, our results are consistent with a model where muscle activation is generated via proprioception with an approximately fixed phase advance from contraction, and biophysical limitations of the muscle yield the forces observed in various viscosities.

## 4. Discussion

### 4.1. GCaMP3 detects body wall muscle activity in freely crawling and swimming worms

By combining automated worm tracking with calcium imaging, we have developed a system to investigate the relationship between muscle activity and body curvature in unrestrained worms during crawling and swimming locomotion. We find, as others have [5,40], that ventral muscle activity is high during ventral body bends, dorsal muscle activity is high during dorsal bends and that ventral muscle activity is out-of-phase with dorsal muscle activity. These relationships apply along the length of the body.

This observed pattern of body wall muscle activity is consistent with the cross-inhibition model of *C. elegans* locomotion that is believed to be coordinated by the D class motor neurons [41,42]. These neurons are postsynaptic to excitatory A and B class motor neurons and have their outputs onto the diametrically opposite muscles [42]. In this way, they are proposed to function in a negative feedback loop in which motor neuron activity causing muscle contraction on one side of the body results in inhibition and relaxation on the other side.

### 4.2. Muscle activation is phase-shifted from predicted force generation

After correcting for phase lags using a variety of different measurements and assumptions, we found that muscle activation, as reported by the GCaMP3 calcium indicator, occurs at an approximately fixed phase ahead of muscular contraction (45° ahead of peak curvature according to the best-fit method), at least for animals swimming in fluids of a wide range of viscosities. In particular, whether we used estimates from electrophysiology or vulval muscle recordings, quantified filtering by fitting to amplitude reductions or applied a simple decay-plus-sparsity condition, the phase advance of muscle activation depended only modestly on viscosity, despite large changes in external force and in period of the swim cycle. The phase advance agrees qualitatively with hysteresis in curvature-calcium plots [40] and observations of curvature-calcium relationships [5] in the earlier work.

However, there are good reasons to believe that the phase advance of force generation does depend strongly on viscosity [1]. We found that this difference probably arises from the inability of muscle to generate equal force at all velocities and lengths. Because the increase in swim frequency as viscosity is lowered outpaces the decrease in curvature, the velocity of individual muscle cells approximately doubles as a worm goes from crawling to swimming in water (data not shown). Although the force–velocity relationship for *C. elegans* muscle is not known, a variety of choices of parameters can explain the observed phase lags to within the uncertainty of our measurements.

### 4.3. Mechanisms for generation of muscle activation at a constant phase-shift

Although our data clearly support an approximately constant-angle phase advance of activity ahead of (i.e. posterior to) maximal curvature, the precise value of the advance depends on the assumptions we make about the filtering properties of the GCaMP3 calcium indicator and cytosolic calcium. We favour the best-fit results of approximately 45° both because it is by construction the best fit to the amplitude data, which otherwise would require some additional explanation, and because there are reasons to doubt that each of the other methods is ideal: vulval muscle is a different tissue, the electrophysiology was performed in dissected animals with buffers that only roughly approximate extracellular and intracellular conditions, and sparsity while conceptually appealing is a rather ad-hoc constraint.

What neural mechanisms could lead to the generation of a constant-angle phase advance? One possibility is that the B-class motor neurons, which are sensitive to total curvature [39] are also sensitive to rate of change of curvature. B-class motor neurons have long posterior-directed processes [42,43] with no known function. Our results indicate that a weak current activated by increasing stretch located at the posterior tip of the process would provide the neuron with input matching the phase of the muscle activation; no such current has been observed [39] but it is unclear that it has been definitively tested. To examine this and other possibilities, further examination of motor neuron activity is necessary.

If proprioception and mechanical constraints at least in principle can account for the range of gaits observed and different viscosities, how does one account for the inappropriate locomotory patterns during optogenetic stimulation of dopamine and serotonin neurons [9]? Our data are consistent with a modulated single-gait model and do not show a strong signature of two distinct gaits. However, this does not preclude a critical role for neuromodulators in shifting where along the gait spectrum the worm's locomotion lies. For instance, neuromodulation may be important for setting the frequency and amplitude of the waveform via head bending, and may also be required to, for instance, tune the relative strengths of proprioceptive feedback and command neuron input. Misregulation of frequency, amplitude or feedback would be expected to cause sizable changes in locomotion pattern.

Finally, it should be noted that data interpretation and force modelling rely on a number of untested assumptions and simplifications. Detailed biophysical models with neural feedback [12] could be extended to test and explore our results in the regime of high-viscosity swimming. Additional experiments that more directly monitor neural activity and muscle activation would also be of great value. Nonetheless, constant phase activation generated via proprioception and shaped by muscle force–velocity and force–length relationships provides a simple and biomechanically plausible mechanism for force generation during locomotion.

## Supplementary Material

Supplementary Figures and Methods

## Data accessibility

Data collected for this study are available at http://dx.doi.org/10.6084/m9.figshare.1213869.
