# Supplementary material for: A consistent muscle activation strategy underlies crawling and swimming in Caenorhabditis elegans
Source: J R Soc Interface. 2015 Jan 6;12(102):20140963. doi: 10.1098/rsif.2014.0963 (PMC4277086; doi:10.1098/rsif.2014.0963)
Supplement: Supplementary Figures and Methods [file rsif20140963supp1.pdf]

Supplementary Methods

Dorsal and ventral curvature were obtained from fluorescence images by taking the brightest point along a ray perpendicular to the midline on dorsal and ventral side, respectively; these points were then smoothed with a constant-speed spline. Curvature was measured by taking points 4% of the body length ahead and behind the point of interest. Dorsal and ventral curvature was ascribed to the position along the body corresponding to the closest midpoint; a point 50% along the midline of the body was chosen as standard (other positions were inspected and appeared to show qualitatively similar relationships). To detect phase shifts between dorsal and ventral curvature, we plotted them against each other, subtracted the best-fit quadratic, and examined the residual spread along the minor axis for time-aligned and time-shifted plots. The best shift was estimated by the minimum point of a quadratic fit to five time shifts from -2 to +2 frames. Little to no overall phase shift was observed, so we examined phase shifts between contraction and elongation (i.e. phase disparity across the width of the body) by performing the same analysis on the two halves of the graphs. If contraction were phase-shifted relative to elongation, the two halves would show equal but opposite phase shifts, so we computed the difference between phase shifts as a measure of overall asynchrony.

Supplementary Figure Legends

Supplementary Figure 1. Calcium activity kymographs during crawling.

(A) Kymograph of body curvature (identical to Figure 2B). (B-C) Corresponding calcium activity. Red bands represent high GCaMP fluorescence, and blue bands represent low GCaMP fluorescence. (B) Ventral muscle calcium levels. (C) Dorsal muscle calcium levels.

Supplementary Figure 2. Phase advance of muscle activation along body.

Uncorrected phase relationships between curvature and GCaMP3 fluorescence as a function of body position (from segments 5 to 95) during crawling. Two independent wild-type data sets are shown (n = 6 and n = 5 animals respectively). Error bars indicate SEM.

Supplementary Figure 3. Synchrony of dorsal and ventral curvature.

(A) Examples of dorsal and ventral curvature for worms swimming on 0-50% dextran, for worms crawling, and for numerically generated noisy sinusoidal data where contraction precedes elongation by a maximum of two frames (66 ms). (B) Estimated asynchrony in milliseconds between dorsal and ventral curvature for each condition in (A). Note that all true data is synchronous to within 20 ms, and that approximately 66 ms asynchrony is recovered for the fake data.

Figure S1

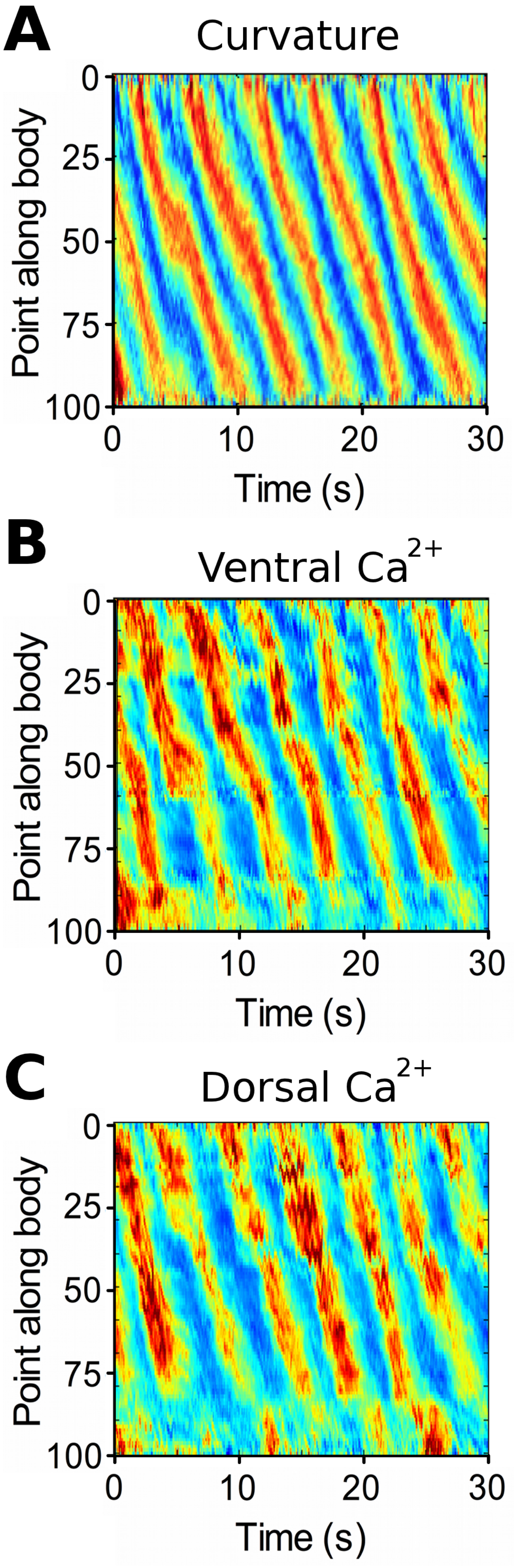
A
Curvature
0
25
50
75
100
Point along body
0
10
20
30
Time (s)
B
Ventral Ca2+
0
25
50
75
100
Point along body
0
10
20
30
Time (s)
C
Dorsal Ca2+
0
25
50
75
100
Point along body
0
10
20
30
Time (s)

Figure S2

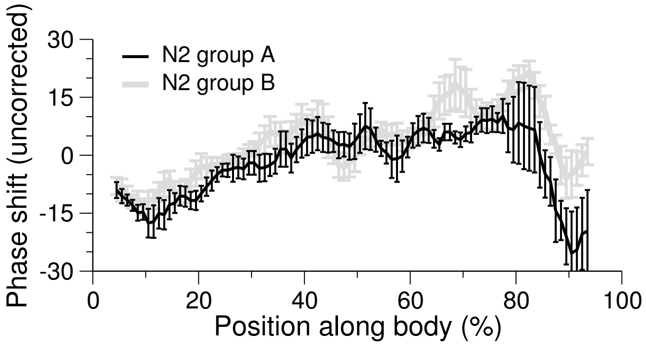

N2 group A
N2 group B
Phase shift (uncorrected)
30
15
0
-15
-30
0
20
40
60
80
100
Position along body (%)

Figure S3

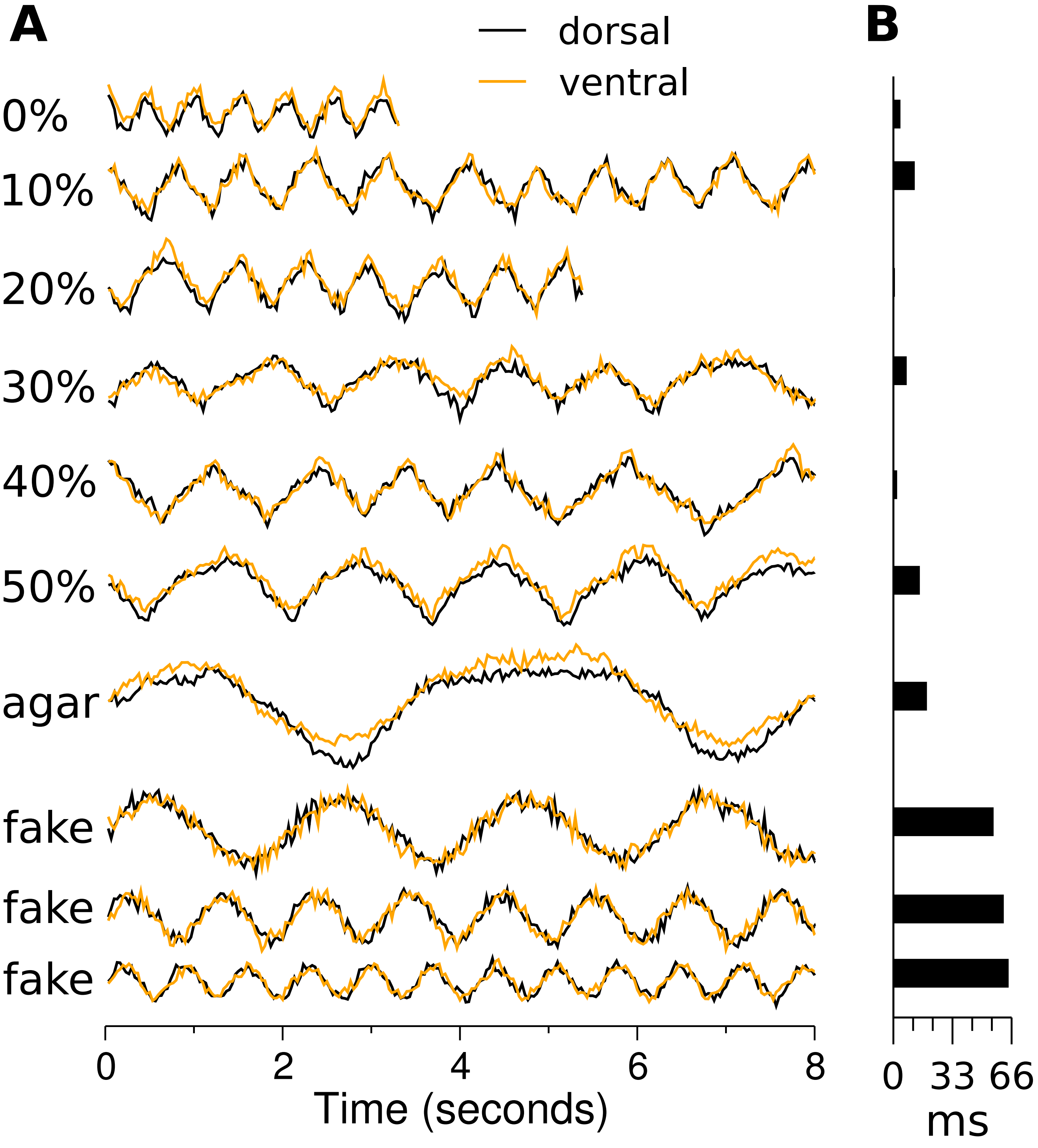
A
B
dorsal
ventral
0%
10%
20%
30%
40%
50%
agar
fake
fake
fake
0
2
4
6
8
Time (seconds)
0
33
66
ms
